# Aloin Inhibits Müller Cells Swelling in a Rat Model of Thioacetamide-Induced Hepatic Retinopathy

**DOI:** 10.3390/molecules23112806

**Published:** 2018-10-29

**Authors:** Eunsoo Jung, Junghyun Kim

**Affiliations:** 1 Laboratory of Toxicology, Research Institute for Veterinary Science and College of Veterinary Medicine, Seoul National University, Seoul 08826, Korea; ozz79@snu.ac.kr; 2 College Department of Oral Pathology, School of Dentistry, Chonbuk National University, Jeonju 54896, Korea; 3 Korea Institute of Oriental Medicine, Daejeon 34054, Korea

**Keywords:** aloin, hepatic retinopathy, Müller cell, swelling

## Abstract

Swelling of retinal Müller cells is implicated in retinal edema and neuronal degeneration. Müller cell swelling is observed in patients with liver failure and is referred to as hepatic retinopathy. In the present study, we evaluated the effects of aloin, an anthraquinone-C-glycoside present in various *Aloe* species, on Müller cell dysfunction in a rat model of thioacetamide (TAA)-induced hepatic retinopathy. Experimental hepatic retinopathy was induced by three injections of TAA (200 mg/kg/day, intraperitoneal injection) for 3 days in rats. After the last injection of TAA, aloin (50 and 100 mg/kg) was orally gavaged for 5 days. The effects of aloin on the liver injury, serum ammonia levels, Müller cell swelling, glial fibrillary acidic protein (GFAP) expression, and gene expression of Kir4.1 and aquaporin-4 were examined. TAA-injected rats exhibited liver failure and hyperammonemia. In the TAA-injected rats, Müller cell bodies were highly enlarged, and GFAP, an indicator of retinal stress, was highly expressed in the retinas, indicating a predominant Müller cell gliosis. However, administration of aloin suppressed liver injury as well as Müller cell swelling through the normalization of Kir4.1 and aquaporin-4 channels, which play a key role in potassium and water transport in Müller cells. These results indicate that aloin may be helpful to protect retinal injury associated with liver failure.

## 1. Introduction

The liver is an important vital organ and has the greatest capacity to eliminate harmful toxins and produce useful substances essential to the human body [1]. Liver cirrhosis is known as the end-stage of liver failure and results in hepatic dysfunction, abnormal metabolism, and death [2]. Hepatic encephalopathy is a neurological disorder that presents in patients with liver insufficiency. Hepatic dysfunction leads to a decline in intellectual capacity, delirium, signs of gliopathy, and the enlargement of brain astrocytes [3]. Hyperammonemia resulting from liver failure also cause morphological alterations in Müller cells in the retina. This is called hepatic retinopathy [4,5]. The Müller cell is one of the retinal glial cells and contributes to a functional link between neurons and vessels in the retinal tissues. In the normal retina, Müller cells help to maintain neuronal activity and support the blood-retinal barrier. However, under several disease conditions, gliotic changes of Müller cells may lead to retinal degeneration and edema [6]. Müller cells also control ion balance [7] and maintain homeostasis of water content in the retinal tissues [8]. When this supportive activity of Müller cells is altered under disease conditions, such as hepatic retinopathy, diabetes-induced retinal dysfunction, and age-related macular degeneration, Müller cells contribute to a dysregulation of metabolism and water homeostasis, resulting in the development of retinal edema and neuronal cell injury [9].

Plants in the genus *Aloe* have been used for medicinal purposes over many centuries worldwide. Aloe gel is widely used and sold worldwide in various cosmetic, health care, and therapeutic products [10]. *Aloe ferox* (also known as the Cape Aloe) is widespread in southern Africa. *A. ferox* has been traditionally used for therapeutic purposes for burns, skin cancer, gastrointestinal diseases, inflammation, and so on [11,12]. Today, *Aloe* is reputed for its treatment of constipation [13], antioxidant properties [14], anti-prediabetes/metabolic syndrome effect [12,13], re-epithelialization of corneal tissue [15,16], and reduction of liver injury [17]. Aloe gel inhibited liver damage in experimental diabetic rats [18]. Aloe extract decreased naphthoquinone-induced toxicity in rat hepatocytes [19]. Intraperitoneal injections of aloe emodin protected against carbon tetrachloride-induced acute liver injury and reduced the levels of serum alanine aminotransferase (ALT) and aspartate aminotransferase (AST) [20]. These previous in vitro and in vivo data suggest that aloe extract possesses a hepatoprotective effect.

**Figure 1:**
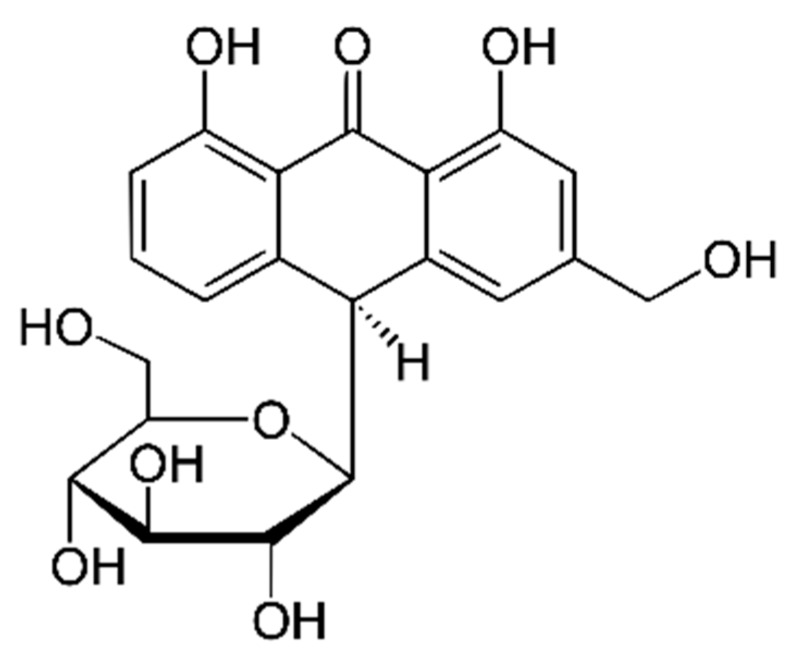
Chemical structure of aloin.

Aloin is an anthraquinone-C-glycoside present in various *Aloe* species (Figure 1). Cui et al. reported that aloin had a protective effect on alcoholic liver disease in mice [21]. Aloin inhibited neuronal cell death after cerebral ischemia [22]. According to these previous reports, it is hypothesized that aloin may have a potent inhibitory effect on hepatic retinopathy. Although extensive studies have been conducted on the effects of the extracts of *Aloe* species and its bioactive compounds on various diseases, the effect of aloin on hepatic retinopathy has not been explored. To elucidate this issue, we investigated the therapeutic effect of aloin on the development of hepatic retinopathy using a rat model of thioacetamide (TAA)-induced acute liver injury. We also determined the effects of aloin on Müller cell response and the expression of aquaporin-4 (glial water channel) and Kir4.1 (potassium channel) in hepatic retinopathy.

## 2. Results

### 2.1. Histopathological Changes in Liver

**Figure 2:**
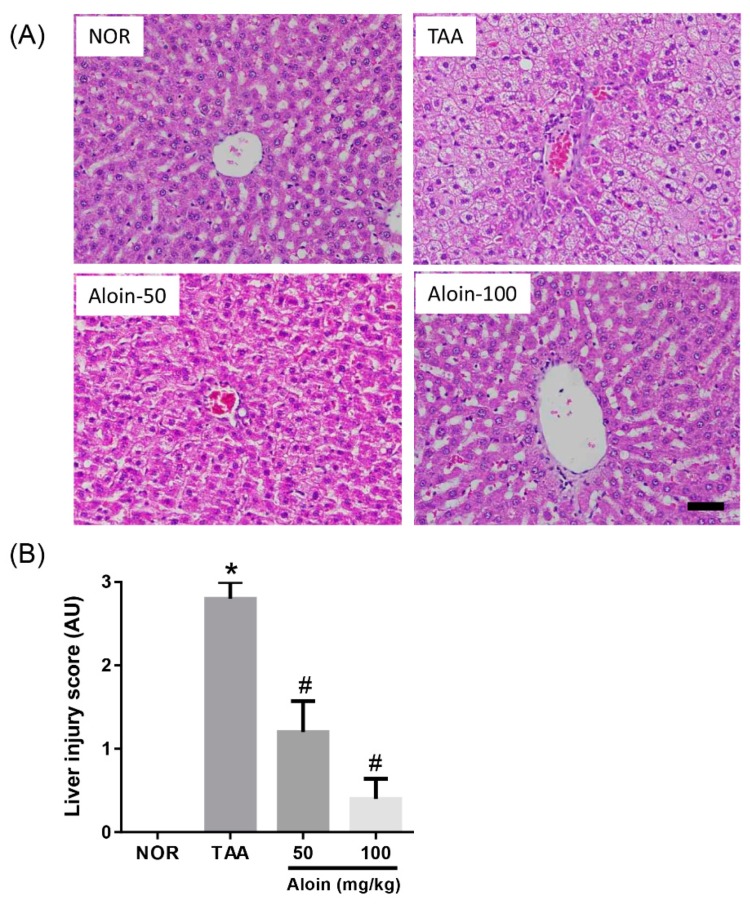
Effect of aloin on liver injury induced by thioacetamide (TAA). (**A**) Histopathological changes in the liver. Liver tissue sections were stained with hematoxylin and eosin. Scale bar = 50 µm. (**B**) Liver injury scores. Values in the bar graphs represent the mean ± SEM, *n* = 7. * *p* < 0.05 vs. normal (NOR) control rats, *# p <* 0.05 vs. TAA-injected rats. AU: arbitrary unit.

Histopathological examination was performed. by hematoxylin and eosin (H&E) staining and liver injury scoring. The livers of normal healthy animals had a normal histological appearance, and hepatocytes showed no degeneration or necrosis. In the TAA group, 200 mg/kg TAA treatment caused acute focal necrosis and vacuolization in some hepatocytes with mild inflammatory cell infiltration (Figure 2A). However, the observed liver injury induced by TAA injection was ameliorated by the treatment with aloin. Similarly, the liver injury score of the TAA-injected rats was markedly increased compared with the normal rats, and rats administered with aloin had significantly decreased liver injury scores (Figure 2B).

### 2.2. Serum Ammonia Levels

**Figure 3:**
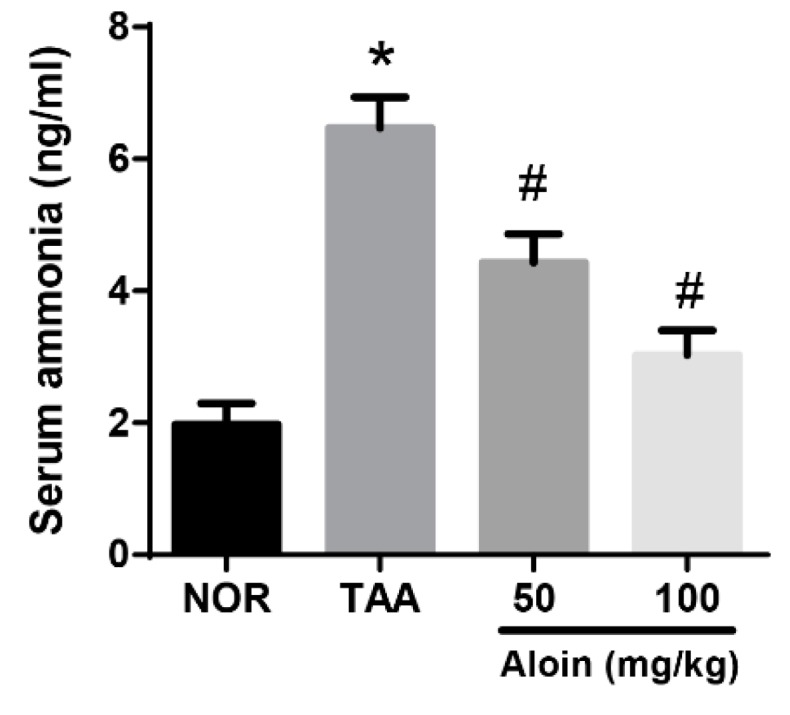
Effect of aloin on serum ammonia levels. Values in the bar graphs represent the mean ± SEM, *n* = 7. * *p* < 0.05 vs. normal control rats, # *p* < 0.05 vs. TAA-injected rats.

Serum biochemical values were assessed in rats. As shown in Figure 3, rats receiving TAA had dramatically increased blood ammonia levels compared with the NOR group (1.98 ± 0.78 vs. 6.47 ± 1.15 ng/mL, *p* < 0.01). Serum ammonia levels were dose-dependently reduced in rats with aloin treatment (4.43 ± 1.05 and 3.03 ± 0.91 ng/mL, respectively).

### 2.3. Müller Cell Swelling

**Figure 4:**
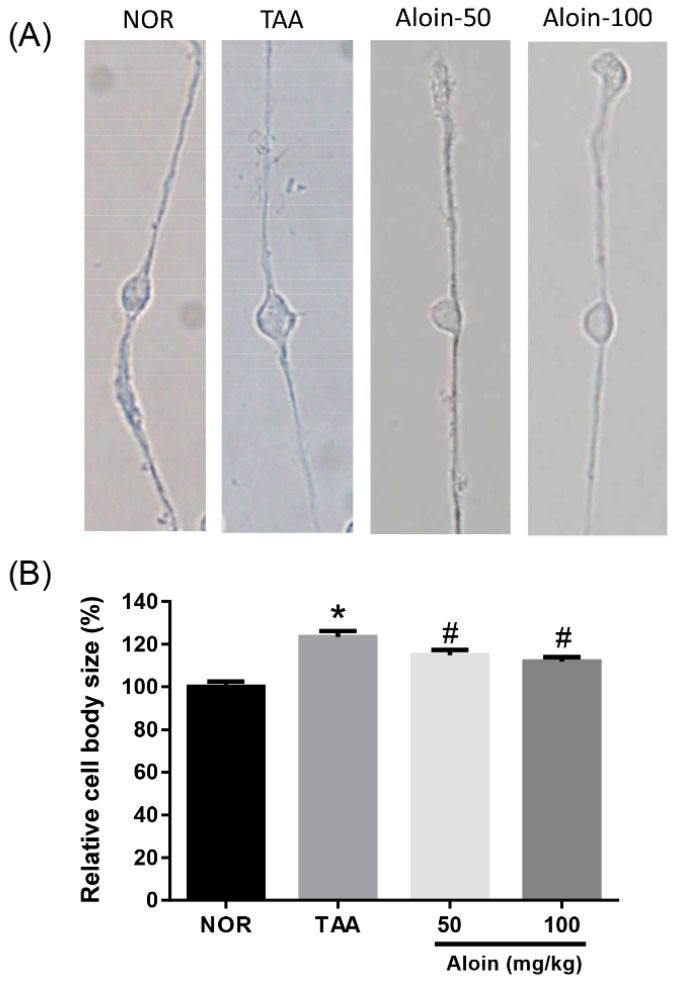
Effect of aloin on Müller cell swelling. (**A**) Enzymatically dissociated Müller cells. (**B**) Quantitative analysis of Müller cell body size. Values in the bar graphs represent the mean ± SEM, *n* = 7. * *p* < 0.05 vs. normal control rats, # *p* < 0.05 vs. TAA-injected rats.

The swelling of Müller cell bodies was evaluated in enzymatically dissociated Müller cells. As shown in Figure 4, TAA-injected rats with liver injury showed significant swelling of Müller cell bodies (*p* < 0.01). The liver failure-evoked swelling of Müller cell bodies was significantly prevented in the TAA-injected rats with aloin treatment (*p* < 0.01). The inhibitory effect of aloin on the swelling of Müller cell bodies was dose-dependent.

### 2.4. Müller Cell Activation

**Figure 5:**
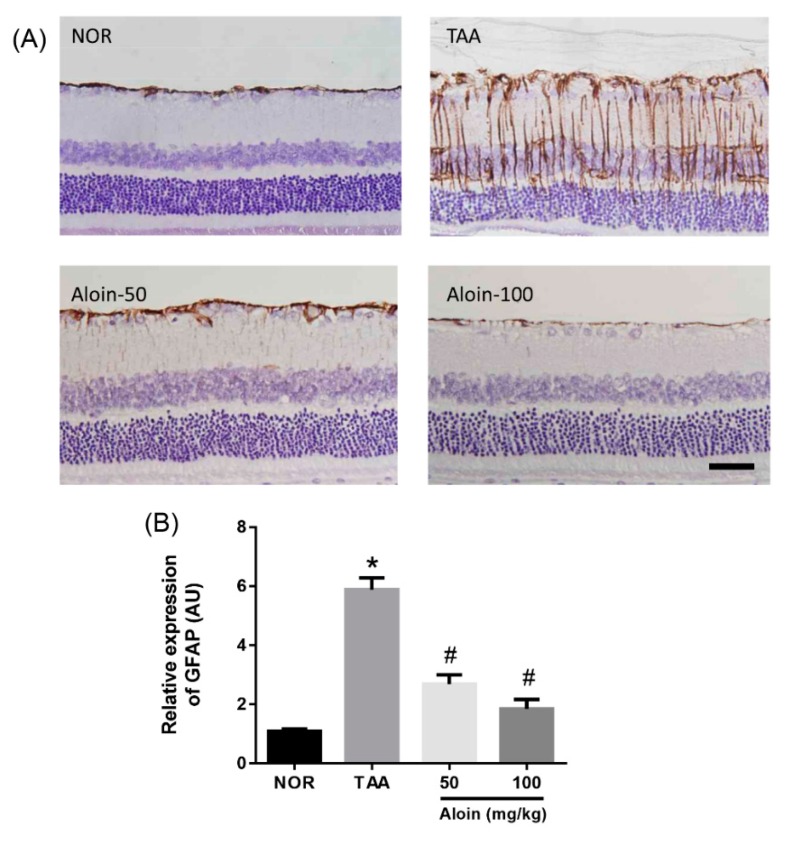
Effect of aloin on glial fibrillary acidic protein (GFAP) expression. (**A**) Immunohistochemical staining for GFAP in the retinal tissues sections. Scale bar = 50 µm. (**B**) Quantitative analysis of signal intensity. Values in the bar graphs represent the mean ± SEM, *n* = 7. * *p* < 0.05 vs. normal control rats, # *p* < 0.05 vs. TAA-injected rats.

Gliotic altered Müller cells have increased GFAP immunoreactivity under pathological conditions [23]. We further investigated the expression level of the GFAP protein in the TAA-injected rats by immunohistochemistry. Figure 5 shows that in normal control retinal section, GFAP proteins were seen mainly at astrocytes in the ganglion cell layers and the end feet of Müller cells. In TAA-injected rats, the expression of the GFAP protein in Müller cells was highly increased, which was linked to liver injury. However, aloin treatment dose-dependently reduced GFAP expression in Müller cells (*p* < 0.01).

### 2.5. Retinal Gene Expression of Kir4.1 and Aquaporin-4

**Figure 6:**
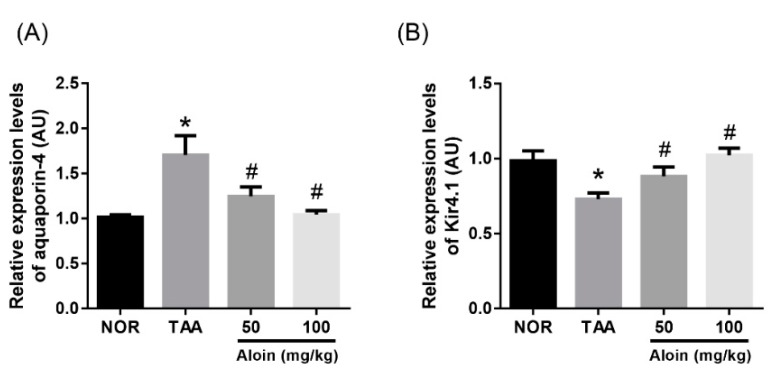
Effect of aloin on aquaporin-4 and Kir4.1 mRNA expression. Real-time PCR analysis of (**A**) aquaporin-4 and (**B**) Kir4.1 mRNA levels. The data are shown as the mean ± SEM, *n* = 7, * *p* < 0.05 vs. normal control rats, # *p* < 0.05 vs. TAA-injected rats.

We examined changes in the gene expression of potassium and water channels involved in Müller cell swelling. Kir4.1 and aquaporin-4 are implicated in potassium and water transport in Müller cells, respectively. To assess this, we analyzed the RNA by real-time PCR, and the expression of aquaporin-4 and Kir4.1 was quantified. Increased aquaporin-4 and decreased Kir4.1 gene expression was observed in TAA-injected rat retinas (Figure 6). The downregulation of potassium channels (Kir4.1) and upregulation of water channels (aquaporin-4) in the retina might contribute to impairment of the Müller cell osmoregulation. These changes of gene expressions were markedly reversed by the treatment with aloin in a dose-dependent manner (*p* < 0.01).

## 3. Discussion

In patients with liver failure, a retinal disorder is observed, which is referred to as hepatic retinopathy [24,25]. Swelling, vacuolization, and necrosis of Müller cells are characteristic features of hepatic retinopathy [26]. Müller cells have a central role in the survival of photoreceptors and neurons, modulate the immune response in the retina, and stabilize the structure of the retinal tissue [27]. Thus, Müller cells have been considered potential therapeutic targets to inhibit these retinal degenerative diseases [28]. In this study, we showed that aloin, a bioactive compound present in various *Aloe* species, prevented Müller cell swelling induced by liver cirrhosis in a rat model with TAA-induced hepatic retinopathy.

The hallmark of hepatic retinopathy is the swelling of Müller cells. TAA has been widely used to produce liver cirrhosis in experimental animals similar to human cirrhosis [29]. The retinas of rats with liver insufficiency induced by TAA were characterized by enlargement of the Müller cell body [30]. Although the precise pathogenic mechanisms leading to this structural change of the Müller cells are unknown, this morphological alteration of Müller cells in the TAA-injected rats is quite resemblant of human hepatic retinopathy. In our study, the rats injected with TAA also showed severe hepatic necrosis, hyperammonemia, and increased Müller cell volume. We investigated the therapeutic activities of aloin for the treatment of hepatic retinopathy using this animal model.

Müller cells are a type of retinal glial cells. Ammonia is toxic to glial cells and leads to glial swelling [31]. Gliosis is a cellular reaction to protect the tissue from further injury and leads to the morphological, biochemical, and physiological alteration of glial cells [32]. Gliotic alterations of Müller cells include cellular hypertrophy and proliferation [33]. Upregulation of GFAP is known to be a very sensitive early indicator of retinal stress [23]. In the present study, we showed that GFAP was highly expressed in the retinas of the TAA-injected rats with hepatic retinopathy, indicating a predominant Müller cell gliosis. Aloin treatment dramatically inhibited the expression of GFAP in the TAA-injected rats. These results indicate that aloin has preventive effects on Müller cell gliosis.

A major role of Müller cells is to maintain ion and water homeostasis in the retinal tissues [7,8]. The fluid absorption is mediated by intramembranous water transport via aquaporins. These water transporters promote bidirectional water flow across membranes [34]. Müller cells have transmembrane aquaporin-4 channels [8]. This water transport by aquaporin-4 is tightly coupled to fluxes of osmolytes, in particular of potassium ions [35]. When a neuron is activated, potassium ions are released from this neuron. In order to prevent neuronal hyperexcitation by excessive potassium, Müller cells take up an excess of potassium ions from the extracellular space in the retina and release a similar amount of potassium into the blood and the vitreous [6]. This spatial buffering of the potassium concentration is mediated predominantly by inwardly rectifying potassium (Kir) channels localized in Müller cell membranes. The Kir4.1 channels and aquaporin-4 channels are colocalized in Müller cells around retinal vessels [36]. When Kir4.1 channels were downregulated in various retinal diseases, Müller cells were osmotically swollen [37,38,39]. Because gliotic altered Müller cells with decreased potassium conductance induced neuronal degeneration and retinal edema, the maintenance of the Kir4.1 channels might contribute to the inhibition of retinal injury and the maintenance of regular neuronal activity.

In the present study, increased aquaporin-4 and decreased Kir4.1 gene expression was observed in TAA-injected rat retinas. Our data suggest that the swelling of Müller cells may be associated with increased water absorption in response to a transmembranal osmotic imbalance, which is induced by the decreased extracellular release of potassium ions after downregulation of Kir4.1 channels. The osmotic swelling of Müller cell bodies is inhibited in the administration of aloin. This swelling-inhibitory effect of aloin may be mediated by the upregulation of the retinal expression of Kir4.1 and maintenance of the potassium currents. Further experiments using immunohistochemical staining are needed to more precisely determine the subcellular localization and expression pattern of Kir4.1 and aquaporin-4 channels in Müller cells.

TAA has been used to induce acute liver injury in rats [40]. TAA administration increased the levels of inflammatory cytokines, such as TNF-α and IL-6. These changes caused liver damage [41]. Aloin protected chronic alcoholic liver injury by attenuating oxidative stress and the inflammatory response [21]. These reports suggest that the aloin might inhibit TAA-induced liver injury through its anti-inflammatory activity. Interestingly, aloin also successfully inhibited TAA-induced liver injury and hyperammonemia. This result suggests that the amelioration of hyperammonemia by the administration of aloin contributed to protecting against Müller cell swelling independently of its antiedematous effect. However, Lucas and Newhouse reported that administration of ammonia did not induce acute toxicity in the retina [42]. Izumi et al. also reported that when a retinal segment was incubated with 1 mM ammonia for 60 min, there was little effect on Müller cells [43]. Although a retina-specific inhibitory effect of aloin was not shown in this study, we demonstrated that aloin led to the reduction of gliotic alterations of Müller cells and prevented Müller cell swelling mediated by the downregulation of Kir4.1 channels. These findings suggest that aloin may have a potent direct antiedematous effect on Müller cells.

In conclusion, our study demonstrated that aloin can surely suppress liver injury as well as the Müller cell swelling that is attributable to the normalization of Kir4.1 channels. Aloin may be helpful to protect against retinal injury associated with liver failure. Further research. is required to determine its direct inhibitory roles in Müller cell swelling.

## 4. Experimental Section

### 4.1. Animals and Experimental Design

Male 6-week-old Sprague-Dawley rats (Orient Bio, Seoul, Korea) were randomly divided into four groups (*n* = 7) as follows: (1) normal control rats, (2) TAA-treated rats, (3) TAA-treated rats treated with aloin (50 mg/kg body weight), and (4) TAA-treated rats treated with aloin (100 mg/kg body weight). To induce hepatic retinopathy, the rats received three intraperitoneal injections of TAA (200 mg/kg, Sigma, St. Louis, MO, USA) for 3 days at 24 h intervals [30]. In the control rats, an equal volume of sterilized saline was injected for 3 days. After the last injection of TAA, aloin (50 and 100 mg/kg, Sigma, St. Louis, MO, USA) was orally gavaged for 5 days. All animals were sacrificed one day after the last administration. All procedures performed on the animals were approved by our Institutional Animal Care and Use Committee (IACUC; approval no. 15-008).

### 4.2. Evaluation of Liver Injury

At necropsy, blood samples were drawn from cardiac puncture. Serum ammonia levels were determined using an Ammonia Assay Kit (Abcam, Cambridge, MA, USA). For histopathological analysis, liver tissue was fixed into 10% formalin for 24 h and embedded in paraffin. Liver tissue sections were stained with hematoxylin and eosin (H&E). The degrees of inflammation and fibrosis were scored with a scale of 0–3 in a double-blind fashion according to a previously reported protocol [44].

### 4.3. Isolation of Rat Retinal Müller Cells

At necropsy, left eyes were enucleated. The whole retina was carefully isolated under a dissecting microscope. Retina was immersed in DPBS containing 1 mg/mL papain (Sigma, St. Louis, MO, USA) for 30 min at 37 °C. After removal of the digestion media, the retina was washed twice with papain-free DPBS. The retinal tissue was mechanically dissociated in papain-free DPBS solution (2 mL). After 10 cycles through the pipette, the Müller cell-rich suspensions were then spread on glass slides; the tissue fragments were permitted to settle for 10 min. Thereafter, 4% paraformaldehyde was added to the suspensions and the cells were fixed for 5 min. The Image J. software (NIH, Bethesda, MD, USA) was used to measure the Müller cell soma area. The morphometric analysis of 50 randomly selected cells was performed for each rat.

### 4.4. Immunohistochemical Staining

At necropsy, right eyes were enucleated and fixed in 4% paraformaldehyde. After fixation, corneas were removed and embedded in paraffin. Paraformaldehyde-fixed and paraffin-embedded retinal sections were deparaffinized in xylene, rehydrated, and treated with 1% H_2_O_2_ in methanol. After PBS washing, sections were incubated with anti-glial fibrillary acidic protein (GFAP) antibody (Abcam, Cambridge, MA, USA) for 1 h at 37 °C. Signal was visualized by 3,3′-diaminobenzidine tetrahydrochloride. The signal intensity was calculated using ImageJ. software (NIH, Bethesda, MD, USA).

### 4.5. Real-Time PCR

Frozen retinal samples were weighed and the total RNA was extracted using TRIzol reagent (Invitrogen, Waltham, MA, USA). Real-time quantitative RT-PCR was conducted according to a previously described protocol [45]. The primer sequences for aquaporin-4 were: forward 5′-TCT CAG TGG GAA ATG TAG CC-3′ and reverse 5′-TGT CTG CAG TGC TGC TAT AA-3′. The primer sequences for Kir4.1 were: forward 5′-CTA GTG GCT CCA GGA ATA CG-3′ and reverse 5′-GCA TGT CAA TGA AGG TCG TC-3′. The primer sequences for ß-actin were: forward 5′-AAA GAG AAG CTG TGC TAT GT-3′ and reverse 5′-TGT AAA ACG CAG CTC AGT A-3′. The mRNA levels of aquaporin-4 and Kir4.1 were determined using the iQ5 optical system software (Bio-Rad Laboratories, Inc., Hercules, CA, USA).

### 4.6. Statistical Analysis

Data in all tables and figures were presented as the mean ± standard error of the mean (SEM). GraphPad Prism v6.0 software (GraphPad Software, Inc., La Jolla, CA, USA) was used to analyze quantitative data. Significant differences were assessed by one-way analysis of variance (ANOVA) followed by Tukey’s multiple comparison test. Because the scoring data for liver injury were nonparametric, comparative analysis was conducted using the Kruskal-Wallis test. Differences were considered statistically significant at *p* < 0.05.
